# Oxygen Vacancies in Perovskite Oxide Piezoelectrics

**DOI:** 10.3390/ma13245596

**Published:** 2020-12-08

**Authors:** Marina Tyunina

**Affiliations:** 1 Microelectronics Research Unit, Faculty of Information Technology and Electrical Engineering, University of Oulu, P.O. Box 4500, FI-90014 Oulu, Finland; marina.tjunina@oulu.fi; 2 Institute of Physics of the Czech Academy of Sciences, Na Slovance 2, 18221 Prague, Czech Republic; tjunina@fzu.cz

**Keywords:** perovskite oxide, ferroelectric, oxygen vacancy, electronic, semiconductor

## Abstract

The excellent electro-mechanical properties of perovskite oxide ferroelectrics make these materials major piezoelectrics. Oxygen vacancies are believed to easily form, migrate, and strongly affect ferroelectric behavior and, consequently, the piezoelectric performance of these materials and devices based thereon. Mobile oxygen vacancies were proposed to explain high-temperature chemical reactions half a century ago. Today the chemistry-enabled concept of mobile oxygen vacancies has been extrapolated to arbitrary physical conditions and numerous effects and is widely accepted. Here, this popular concept is questioned. The concept is shown to conflict with our modern physical understanding of ferroelectrics. Basic electronic processes known from mature semiconductor physics are demonstrated to explain the key observations that are groundlessly ascribed to mobile oxygen vacancies. The concept of mobile oxygen vacancies is concluded to be misleading.

## 1. Introduction

The superb electro-mechanical performance of perovskite oxide ferroelectrics makes these materials the main piezoelectrics. They include the most widely known lead zirconate titanate (PZT), relaxor-based materials (e.g., lead magnesium niobate titanate, or PMNT), lead-free materials based on barium titanate (BTO), potassium niobate (KNO), sodium bismuth titanate, and others. Oxygen vacancies are accepted as the most common mobile point defects, which dramatically influence the ferroelectric behavior and, consequently, the functioning of perovskite oxide piezoelectric materials and devices made of them [1,2,3,4,5,6,7,8,9,10,11,12,13] (see Note). This attitude is widely accepted in materials chemistry and technology. Concurrently, basic solid-state physics and modern first-principles analyses suggest another picture for oxygen vacancies and related phenomena. Surprisingly, the “chemical” and “physical” interpretations exist independently of each other and almost never interact. Here, an attempt is made to discern the “chemical” picture considering physics knowledge.

First, the chemical and physical approaches to oxygen vacancies in perovskite oxide ferroelectrics are briefly overviewed and confronted. Next, a concept of ferroelectric as a wide bandgap semiconductor with in-gap traps is suggested and justified. Basic electronic processes are considered therein. The suggested approach is applied to explain numerous effects, which are ascribed to “chemical” mobile oxygen vacancies. Finally, the concept of mobile oxygen vacancies is inferred as misleading.

## 2. Chemistry

Perovskite oxide ferroelectrics possess the generic chemical formula *A^a^B^b^*O_3_, where the valences of the metal *A* and *B* cations are (*a* + *b* = 6), for instance: Pb^2+^Ti^4+^O_3_, Ba^2+^Ti^4+^O_3_, K^1+^Nb^5+^O_3_, Bi^3+^Fe^3+^O_3_. The stability of the perovskite structure is usually characterized by the Goldschmidt tolerance factor considering the ionic radii of the cations and oxygen anion. Solid solutions of two or more compounds, as well as doping with heterovalent cations, are feasible for a wide range of different perovskite oxides.

The formula, the tolerance factor, and the charge neutrality for solid solutions or doped materials consider perovskite oxides as ionic crystals. From this point of view, a deficit of the positive charge due to heterovalent cationic substitution should be balanced by the corresponding decrement of the negative charge, which is only possible when oxygen anions are removed and, hence, charged oxygen vacancies (V_O_^2+^) are formed. This treatment is based on the Kröger–Vink approach to the point defects in ionic crystals [14]. Having this approach as a starting point, the formation of oxygen vacancies under special oxygen-deficient (or reducing in terms of chemistry) conditions is then thought to create an excess positive charge, which should be balanced by the negative charge of free electrons.

**Figure 1:**
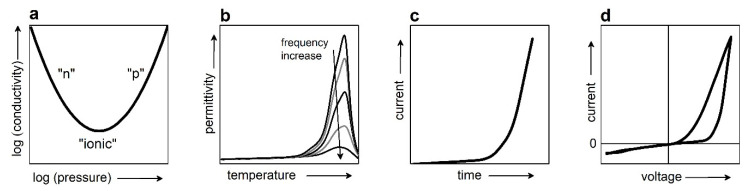
Typical electric behavior of perovskite oxide piezoelectric materials. Schematics of (**a**) V-shaped log–log curve of DC conductivity versus oxygen pressure, (**b**) high-temperature frequency-dependent permittivity peak, (**c**) current during DC degradation, and (**d**) resistive switching. Arrows show directions of parameters increase.

The classical chemical experiment on the formation of oxygen vacancies is performed at the high temperatures of 800–1400 K and varying oxygen pressure (*p*_O2_) from as low as 10^−4^ Pa to atmospheric [1,2,15,16]. During the experiment, the DC electrical conductivity (*σ*) is measured. The “V”-type log–log plot of conductivity versus pressure (Figure 1a) is typically observed. To relate this conductivity to oxygen vacancies, the reactions between oxygen, oxygen vacancies, electrons, holes, and cations, as well as conditions of mass balance and charge neutrality, are taken into account, and mass-action laws are applied. The concentrations of all charged species are calculated as a function of oxygen pressure.

Then, the calculated pressure-dependent concentrations are compared with the measured pressure-dependent conductivity and the “V”-type plot is interpreted as follows: The low-pressure decreasing branch is due to a high concentration of negatively charged species and corresponds to an “*n*”-conductivity. The increasing high-pressure branch is due to the high concentration of positively charged species and corresponds to a “*p*”-type conductivity. There is a transition region between the “*n*”- and “*p*”-branches, where “ionic” conductivity is claimed because the calculated concentrations of the holes and electrons are equal to each other in this range of pressures. In the absence of dopants, the “*n*”-conductivity is ascribed to electrons, which originate from the formation of oxygen vacancies, whereas the charged oxygen vacancies are responsible for the “*p*” and “ionic” conductivities. Thus, the oxygen vacancies are treated concurrently as electronic donors (for the “*n*”-conductivity) and as mobile positive charge carriers (for the “*p*” and “ionic” conductivities).

The concept of oxygen vacancies as mobile charge carriers was coined half a century ago, readily accepted, expanded to arbitrary physical conditions, and transferred to diverse phenomena occurring in ferroelectrics even at low temperatures and normal oxygen pressures. This concept appeared convenient for simple explanations of many effects and is now increasingly popular. For instance, hopping and/or migration of oxygen vacancies are used to explain peaks of the dielectric permittivity (conductivity) often observed at high temperatures (Figure 1b) [6,7,10], an increase of current during DC degradation (Figure 1c) [3,4,5], resistive switching (Figure 1d) [8,11], as well as aging and fatigue [13]. The charged oxygen vacancies are widely believed to be able to migrate with rather high mobility and thus play an important role in ferroelectric behavior, including piezoelectric performance.

## 3. Physics

In contrast to the chemical experiment, physical studies are usually carried out for different samples, whose synthesis suggests either correct oxygen stoichiometry (*AB*O_3_) or oxygen deficiency (*AB*O_3-δ_) therein. Electrical conductivity is measured as a function of temperature, frequency, electric field, and magnetic field. The temperatures are lower than those in the chemical experiment and include as low temperatures as technically available today. The sign (positive or negative) and mobility of charge carriers are directly determined from the Hall and/or Seebeck measurements, whereas the character of conductivity (hopping or band) is concluded from the temperature and frequency dependences.

For both stoichiometric and oxygen-deficient samples, the *n*-type (electronic) conductivity only was found. Conductivity by hopping of small electron polarons was established in stoichiometric ferroelectrics [17,18,19,20,21]. Additionally, electronic band conductivity (i.e., by electrons in the conduction band) was detected for oxygen-deficient samples and/or at high temperatures [22,23,24]. Remarkably, although the electron hopping transport [17] is known for a long time as the chemical experiment is [1], this transport is nearly never considered in studies of ferroelectric piezoelectrics. Despite the experimental physical evidence of the *n*-type conductivity, the concept of oxygen vacancies as mobile charge carriers is unhesitatingly applied to diverse aspects of ferroelectric behavior.

**Figure 2:**
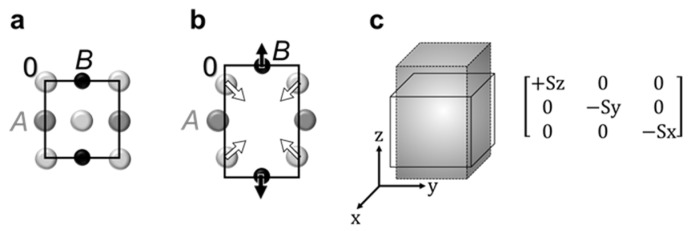
Oxygen vacancy. Schematics of (**a**) regular correct oxygen stoichiometry (*AB*O_3_) perovskite cell, (**b**) distortions around oxygen vacancy, and (**c**) oxygen-vacancy elastic dipole.

It should be stressed that oxygen comprises 60% of atoms in perovskite oxides. State-of-the-art experimental methods of compositional analysis are unable to detect deviations from oxygen stoichiometry with accuracy better than 1 at %. Therefore, the statements on the presence or absence of oxygen vacancies are often equally disputable. One of the most common arguments in support of the presence of oxygen vacancies is an expansion of the crystal lattice, which is associated with vacancies. Indeed, compared to a regular *AB*O_3_ perovskite cell (Figure 2a), there are local lattice distortions around oxygen vacancy V_O_ (Figure 2b). The two *B* ions, nearest to the vacancy, are displaced away from the vacancy, which can be qualitatively understood from simple Coulomb interactions. The *B*-Vo-*B* distance is larger than the *B*-O-*B* distance. This distortion produces an anisotropic elastic dipole (Figure 2c) with positive components (tensile strain) along the *B*-Vo-*B* direction and negative components (compressive strain) in two other orthogonal directions.

**Figure 3:**
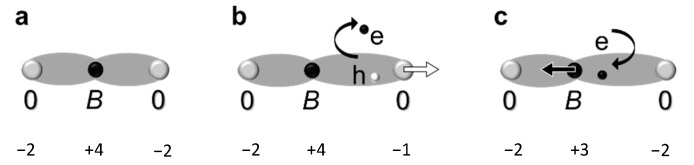
*B*-O lengths. Schematics for (**a**) regular *AB*O_3_, (**b**) hole polaron, and (**c**) electron polaron.

However, despite the strong *B*-Vo-*B* elongation, its effect on lattice expansion is negligibly small because all randomly distributed and differently oriented dipoles almost fully compensate each other on average [25]. For significant oxygen deficit of *δ* = 0.5, the corresponding relative increase of the unit cell volume is smaller than 10^−10^ [25], which is hardly detectable by common X-ray/electron diffraction/scattering experiments. On the other hand, elongations of the *B*-O bonds can be deduced from the data obtained by photoelectron spectroscopy, X-ray absorption spectroscopies, electron energy loss spectroscopy, and others. Do such elongations prove the presence of oxygen vacancies? Not necessarily. The *B*-O elongations can result from the hole polarons (Figure 3b) or electron polarons (Figure 3c) [26,27,28]. The self-trapped polarons can exist in pure undoped stoichiometric materials, and their formation is easier in the presence of heterovalent doping cations. As seen from Figure 3, when an electron is removed, and a hole is localized at the *B*-O bond, the formed hole polaron can be chemically interpreted as the change of the valence state of oxygen with the increased *B*-O distance. Likewise, when an additional electron is localized on the *B*-O site, the chemical interpretation of this electron polaron is the change of the valence state of the *B*-cation (often termed as “oxidation state”). The formation of such polarons is seldom included in spectroscopic analyses and never considered in chemical experiments.

Physical experiments also include important optical investigations (e.g., absorption, luminescence). The seminal experimental and theoretical studies were performed over 60 years ago and demonstrated that ferroelectrics could be treated as semiconductors [29]. The width Δ*E_g_* of the energy gap (between the lowest unfilled band, or conduction band, and the highest filled band, or valence band) is approximately 3 eV in the majority of ferroelectrics [30]. It is worth noting that modern semiconductor materials include such compounds as SiC (Δ*E_g_* ≈ 3 eV), AlN (Δ*E_g_* ≈ 6 eV), GaN (Δ*E_g_* ≈ 3.4 eV), whose bandgaps are comparable with or even wider than those in ferroelectrics. The fundamental approach to ferroelectrics as semiconductors is ignored in the chemical experiment (Figure 1a).

Importantly, progress in computational research, especially first-principles calculations, enabled analysis of such complex materials as perovskite-type metal oxides decades ago [31,32,33,34,35,36]. The first-principles calculations revealed that ferroelectrics are not pure ionic crystals but governed by a significant presence of covalent bonding. The charges of ions are not equal to the nominal valences. The first-principles analyses of oxygen vacancies showed that neutral vacancies are as stable as charged ones and that the vacancy formation depends primarily on the material’s electronic structure [37,38,39,40,41,42].

The chemical approach to ferroelectrics as ionic crystals and to oxygen vacancies as the Kröger–Vink defects is not supported by the physical understanding of these materials. The chemistry-enabled concept of oxygen vacancies as mobile charge carriers is not clearly confirmed by physical evidence. Nonetheless, numerous observations seem fitting to this concept. In the following, it will be demonstrated that pure electronic processes, which are well known from the mature semiconductor physics but mostly disregarded in ferroelectrics, can explain the key observations, which are groundlessly ascribed to mobile oxygen vacancies.

## 4. Wide Bandgap Insulator-Semiconductor Ferroelectric

**Figure 4:**
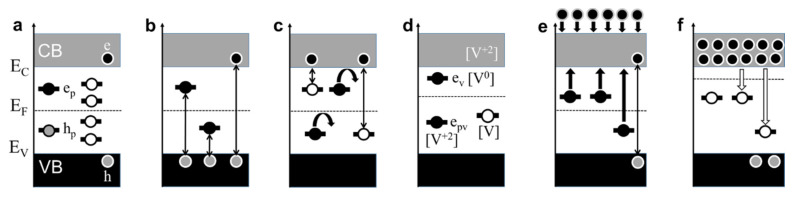
Electronic processes. Schematics of (**a**) electronic band structure, (**b**) excitations from the valence band, (**c**) excitations from in-gap states, (**d**) oxygen-vacancy related in-gap states, (**e**) excitations and injection under applied voltage, (**f**) relaxation to in-gap states.

An ideal defect-free ferroelectric can be treated as an intrinsic wide bandgap semiconductor with the Fermi level in the middle of the gap, separating the valence band (VB) and conduction band (CB) (Figure 4a). At a nonzero temperature, the CB (VB) can contain electrons (holes). Additionally, the possibility for self-trapped electrons and/or holes (polarons) ensures in-gap states in ferroelectrics. Importantly, ferroelectrics possess spontaneous polarization and polarization domains. Domain walls can trap charge carriers [43,44] and deliver either vacant, unoccupied in-gap states or occupied in-gap states as well. Thus, the presence of occupied and free in-gap states is fundamentally characteristic of ferroelectrics. It is important to note that real ferroelectric samples contain point-type and extended defects (unintentional dopants, interstitials, dislocations, twin boundaries, grain boundaries, etc.), as well as surfaces and interfaces with electrodes-all of which can produce additional in-gap traps [43].

Thus, in ferroelectrics, electrons can be excited from the VB to the CB and relax back, and can also be excited to the in-gap states and relax, correspondingly (Figure 4b). The trapped electrons can be excited from the in-gap states, and either may or may not reach the CB (Figure 4c) and relax back, too.

When a neutral oxygen vacancy is formed, it creates occupied in-gap states, typically 0.2–0.5 eV below the CB (Figure 4d) [37,38,39,40,41,42]. The trapped electrons can be excited from these states as in (Figure 4c). A doubly charged vacancy does not create occupied in-gap states and cannot act as an electron donor but can trap electrons [37,38,39,40,41,42]. The electron polaron trapped at the doubly charged vacancy can produce deep occupied in-gap states [41]. Thus, the vacancies (Figure 4d) do not lead to a new specific electronic band structure, which principally differs from that of a regular stoichiometric sample (Figure 4a). An electronic effect of oxygen vacancies is that with increasing concentration of vacancy-related shallow occupied in-gap states, the Fermi level (*E_F_*) uplifts (Figure 4d). It should be stressed that such an uplift can be caused by shallow occupied in-gap states, whose origin is not related to oxygen vacancies at all.

Considering that oxygen vacancies and other defects are immobile and that the charge transport is realized by a combination of hopping of small polarons (*σ_p_*) and band conductivity (*σ_e_* for the electrons in the CB and *σ_h_* for the holes in the VB), the total conductivity *σ* is

(1) $$\sigma =\sigma_{e}+\sigma_{h}+\sigma_{p}$$

The conductivity *σ_i_* due to a carrier having the charge *q_i_*, the mobility *μ_i_*, and whose concentration is *N_i_*, can be generally presented as

(2) $$\sigma_{i}=q_{i}\mu_{i}N_{i}$$

Because of the nearly flat top VB [29,36,37], the hole mobility *μ_h_* is significantly smaller than the electron mobility *μ_e_* in perovskite oxide ferroelectrics:

(3) $$\mu_{h}\ll \mu_{e}$$

Therefore, for the sake of simplicity, the hole conductivity is omitted here. The concentration *N_e_* of electrons is

(4) $$N_{e}=N_{0}exp\left(-\frac{E_{C}-E_{F}}{k_{B}T}\right)$$

where *N_0_* is the density of states in the CB, *k_B_* is the Boltzmann constant, and *T* is the temperature [45]. In the presence of defects, the electron mobility *μ_e_* can be approximated by (5):

(5) $$\mu_{e}\approx \mu_{e0}+\frac{\mu_{00}}{1+\alpha N_{DEF}}$$

where *μ_e_*_0_, *μ*_00_, and *α* are semi-empirical parameters, and the concentration *N_DEF_* describes the defects [46]. The concentration of polarons *N_p_* is temperature-independent, but their mobility *μ_p_* increases with temperature [18,19,20,21,22,23,24]:

(6) $$\mu_{p}=\mu_{p0}exp\left(-\frac{E_{p}}{k_{B}T}\right)$$

where the activation energy *E_p_* is defined by the polaron binding energy and is about ~0.1–0.3 eV [18,22]. For the electron elementary charge *e*, the electrical conductivity is

(7) $$\sigma \approx eN_{0}exp\left(-\frac{E_{C}-E_{F}}{k_{B}T}\right)\left(\mu_{e0}+\frac{\mu_{00}}{1+\alpha N_{DEF}}\right)+eN_{p}\mu_{p0}exp\left(-\frac{E_{p}}{k_{B}T}\right)$$

The conductivity does not directly depend on the concentration of oxygen vacancies but can be affected by the vacancies through the vacancy-enhanced parameters *E_F_*, *N_DEF_*, and *N_p_*.

### 4.1. Pressure-Conductivity Curve

In the chemical experiment (Figure 1a), the temperatures are high, and all trapped electrons are excited to the CB so that the band conductivity dominates (first term in (7)). With decreasing oxygen pressure, oxygen diffuses out of the sample. This process is associated with the formation of defects, including interstitial oxygen and oxygen vacancies, that diminishes the electron mobility (6). Some of the vacancies act as electron donors, which raises the Fermi level. These two tendencies—a decrease of the mobility and uplift of the Fermi level—have opposite effects on the conductivity. For the chemical “*p*” branch of the conductivity, the physical explanation is the decreased mobility of the electrons, whereas the conductivity is *n*-type. For the chemical “*n*” branch of the conductivity, the raise of the Fermi level *E_F_* prevails and overrides the mobility drop. Thus, the non-monotonic “V”-type pressure-conductivity dependence can be explained by semiconductor behavior, where the Fermi level and the mobility vary with the pressure-dependent concentration of oxygen vacancies and interstitials. It is again worth noting that the basic semiconductor approach (7) is never taken into account in the chemical interpretations.

### 4.2. Large Activation Energy for Hopping Conductivity

At low temperatures, hopping transport takes place (second term in (7)). At intermediate temperatures, the trapped electrons are released from the traps, and a fraction of the released electrons are excited to the CB (Figure 4c). With increasing temperature, this fraction grows, which is manifested in the temperature-dependent Fermi level. Then a combined hopping-band conductivity can be described as

(8) $$\sigma \approx e\mu_{e}N_{0}exp\left(-\frac{E_{C}-E_{F}}{k_{B}T}\right)+eN_{p}\mu_{p0}exp\left(-\frac{E_{p}}{k_{B}T}\right)\approx \left[e\mu^{*}N^{*}\right]exp\left(-\frac{E^{*}\left(T\right)}{k_{B}T}\right)$$

Here, *N*^∗^, *μ*^∗^, and *E*^∗^ are the effective parameters (concentration, mobility, and activation energy). The effective energy *E*^∗^ can take the values between the polaron binding energy *E_p_* and half of the bandgap energy Δ*E_g_*/2. Importantly, excitation of the trapped electrons leads to an apparent activation character of the conductivity with the activation energy *E^∗^*, which exceeds the polaron binding energy: E^*^ > *E_p_*. This large activation energy E^*^ > *E_p_* is often extracted from the measured AC conductivity or dielectric permittivity (see Figure 1b) and interpreted as that of “hopping of oxygen vacancies” [6,7,10,11]. As is clear from (8), the thermally stimulated electronic excitations explain the observed E^*^ > *E_p_*.

### 4.3. Current–Voltage Curve

The electronic trap-to-CB excitations can be stimulated not only thermally but also by the electric field. To apply an electric field, a voltage is applied to conducting metal electrodes, between which a ferroelectric layer is sandwiched. The charge carriers are then excited and also injected from the metal into ferroelectric (Figure 4e). The excitation and injection result in the raised Fermi level. Correspondingly, the conductivity can be described using the voltage-dependent effective energy *E^eff^* (*V*):

(9) $$\sigma \approx \left[e\mu^{*}N^{*}\right]exp\left[-\frac{E^{*}}{E^{eff}}\right]$$

**Figure 5:**
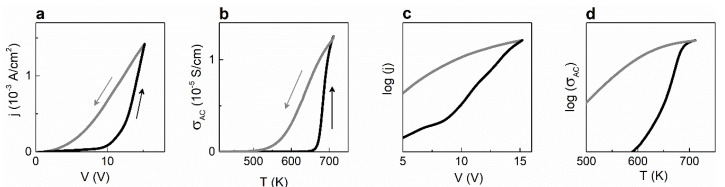
Electric conduction. (**a**,**b**) Linear and (**c**,**d**) semi-log plots of (**a**,**c**) current density while sweeping the DC voltage and (**b**,**d**) AC conductivity during heating and cooling. Arrows show directions of the parameter change in (**a**,**b**). Plots are based on data reported in [47,48].

For a rough linear approximation *E^eff^* = *βV*, the current density *j* is

(10) $$j\approx d^{-1}\left[e\mu^{*}N^{*}\right]exp\left[-\frac{E^{*}}{E^{eff}}\right]V=j_{00}exp\left[-\frac{E^{*}}{\beta V}\right]V$$

where *d* is the thickness (or length) of the sample. The voltage-induced excitation explains the observed exponential increase of current with increasing DC voltage (Figure 5a,c). Remarkably, thermally stimulated excitation also produces a nearly exponential increase of AC conductivity (Figure 5b,d), in agreement with (8).

### 4.4. Current–Voltage Hysteresis

It is worth recalling that the excitation processes are almost instantaneous, with the characteristic times on the scale of femtoseconds, whereas the inverse processes of relaxation to the traps (Figure 4f) are significantly slower, with the characteristic times to nanoseconds, days, months, and even years for certain deep traps (e.g., those associated with domain walls). Therefore, the behavior of current or conductivity after the voltage- or thermally induced excitation is governed by the relatively long relaxation time. Interestingly, the linear current–voltage relaxation curve (Figure 5a) and the linear conductivity-temperature relaxation curve (Figure 5b) point to a linear dependence of the carrier concentration on time because both the voltage and the temperature were changed at constant rates during the measurements. The excitation-relaxation processes explain the current–voltage hysteresis and resistive switching in any ferroelectric, containing traps. The widely employed concept of hypothetical electro-migration of the charged oxygen vacancies is not needed at all.

### 4.5. Degradation

**Figure 6:**
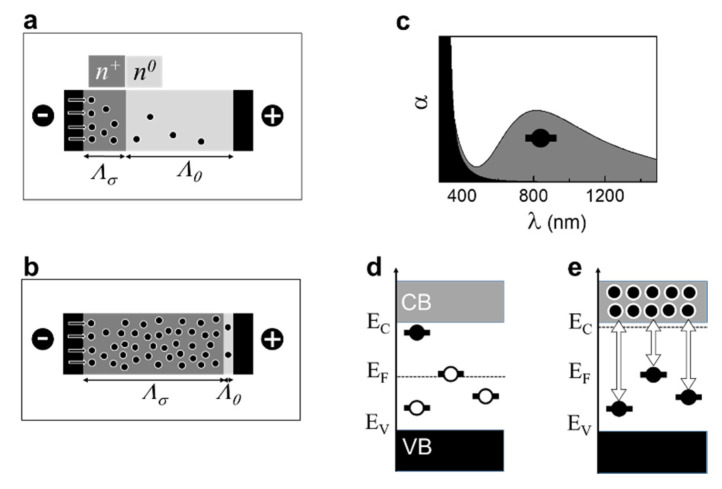
Electronic processes during DC degradation and coloration. (**a**,**b**) Schematics of carrier injection and transport during DC degradation. (**c**) Optical absorption and (**d**,**e**) schematics of (**d**) deep in-gap traps and (**e**) electronic relaxation to these traps.

The “chemical” concept of mobile charged oxygen vacancies (Figure 1a) is believed to be mandatory to explain such phenomenon as DC degradation, which is critical for device applications of ferroelectrics and piezoelectrics [3,4,5]. When a DC voltage is continuously applied to an electrode-ferroelectric-electrode sandwich, an abrupt increase of current is observed at a certain time after the beginning of DC biasing. Such degradation can be explained by the electronic processes as follows: Under the DC voltage, the electrons are excited and, importantly, injected from the electrode (cathode). In the near-cathode region, the injected electrons produce an *n^+^*-type (high carrier concentration) layer, which adjoins the rest of the *n^0^*-type sample (lower carrier concentration) (Figure 6a). This contact presents a forward-biased *n^+^*–*n^0^* junction, where the external DC field lowers the contact potential and enables the movement of the electrons further away from the cathode. When increasing electrons are injected with time during the DC biasing, the *n^+^*–*n^0^* boundary moves towards the anode, the *n^0^*-layer shrinks, the field strength across this layer increases and, finally, causes its breakdown (Figure 6b). Because the injection and motion of electrons do not require oxygen vacancies and/or other defects at all, the DC degradation can occur in a perfect crystal, where carrier injection is allowed.

### 4.6. Coloration

The “chemical” hypothesis of mobile oxygen vacancies is widely employed to explain the decrease of optical transmission, or “coloration” during the chemical experiment and the DC degradation. Typically, an increase of optical absorption is observed in the visible spectral range, i.e., at wavelengths of 550–1000 nm (Figure 6c). These wavelengths correspond to the electronic excitation energies of 1.2–2.2 eV and, respectively, to the deep occupied in-gap states 1.2–2.2 eV below the CB.

The “chemical” interpretation of coloration is the presence and migration of the charged oxygen vacancies. However, the charged vacancies have no in-gap states and cannot be manifested in optical absorption [37,38,39,40,41,42]. Concurrently, neutral vacancies produce shallow in-gap states 0.2–0.5 eV below the CB and thus cannot be manifested in optical absorption in the visible spectral range either.

The coloration effect indicates the formation or filling of the deep in-gap traps. The traps may be associated with dopant cations, defects, domains, etc. (Figure 4a and Figure 6d). Both in the chemical experiment and during the DC degradation, the concentration of free electrons (in the CB) increases (Figure 6e). The abundant electrons enable a high probability for the electronic CB-to-trap relaxation (Figure 6e). The photo-stimulated trap-to-CB excitations are then seen in the absorption spectra (Figure 6c).

### 4.7. Anelastic Relaxation

In addition to electrical and optical behavior, also elastic properties of oxygen-deficient ferroelectrics were argued to prove the hopping and migration of oxygen vacancies [49]. Indeed, because oxygen vacancy produces an anisotropic elastic dipole (Figure 2c), the dipole’s orientation should flip when the vacancy exchanges position with the nearest oxygen ion. This reorientation can be seen in elastic losses, which are deduced from electrically stimulated vibrations of the sample. The activation energies and relaxation times, extracted from this anelastic relaxation, are in the ranges of 0.1–1 eV and 10^−14^ s, correspondingly [49]. Remarkably, the relaxation time is very short, close to that of electronic excitations, and the activation energies are between the polaron binding energy and half of the gap. Notably, vacancy elastic dipole is mainly determined by the Ti–V_O_–Ti elongation. However, it is easy to understand that this elongation depends on the charge state of the vacancy: it is larger for neutral vacancies compared to charged ones. Thus, electronic excitation from the neutral-vacancy in-gap state to the CB is accompanied by changes of elastic dipole tensor (from that of neutral vacancy to that of charged vacancy) and, hence, can be seen in anelastic relaxation. Likewise, other traps may experience elastic changes upon electronic excitations. Interestingly, the temperatures of anelastic relaxation peaks agree well with those for the electronic trap-to-CB excitations in single-crystal SrTiO_3_ [49,50]. This resemblance points to the electronic origin of anelastic behavior.

Thus, the principal experimental observations, which are usually interpreted using hypothetical mobile oxygen vacancies, can be directly explained by electronic processes, which are well known from semiconductor behavior. Importantly, because in-gap traps are essential for the electronic, optical, and elastic behavior of ferroelectrics, the nature of these traps must be better clarified in order to master the ferroelectric and piezoelectric performance.

## 5. Conclusions

In summary, the widely employed concept of oxygen vacancies as mobile charge carriers in perovskite oxide ferroelectrics was debated. The common approach to ferroelectrics as ionic crystals with the Kröger–Vink defects was shown to conflict with the modern physical understanding of these materials. Basic electronic processes known from semiconductors were demonstrated to explain the key observations usually ascribed to mobile oxygen vacancies, including the pressure-conductivity curve, the large activation energy for hopping conductivity, the current–voltage curve, the current–voltage hysteresis, the DC degradation, the coloration, and the anelastic relaxation. The concept of mobile oxygen vacancies was inferred as invalid.

## Note

Publications on mobile oxygen vacancies are extensive and only a few of them are included herein.
